# Chest Computed Tomography for the Diagnosis of COVID-19 in Emergency Trauma Surgery Patients Who Require Urgent Care During the Pandemic: Protocol for an Umbrella Review

**DOI:** 10.2196/25207

**Published:** 2021-05-06

**Authors:** Dylan Griswold, Andrés Gempeler, Gail Rosseau, Neema Kaseje, Walter D Johnson, Angelos Kolias, Peter J Hutchinson, Andres M Rubiano

**Affiliations:** 1 National Institute of Health Research Global Health Research Group on Neurotrauma University of Cambridge Cambridge United Kingdom; 2 Division of Neurosurgery, Department of Clinical Neurosciences Addenbrooke's Hospital University of Cambridge Cambridge United Kingdom; 3 Clinical Research Center Fundación Valle del Lili Cali Colombia; 4 Department of Neurosurgery George Washington University School of Medicine and Health Sciences Washington, DC United States; 5 Surgical Systems Research Group Kisumu Kenya; 6 Schools of Medicine and Public Health Loma Linda University Loma Linda, CA United States; 7 Neuroscience Institute INUB-MEDITECH Research Group El Bosque University Bogota Colombia

**Keywords:** systematic review, broad-evidence synthesis, COVID-19, global health, trauma surgery, evidence-based practice, chest CT, rapid testing, testing, diagnosis, scan, computed tomography, review, antigen, immune system, health care worker, surgery, emergency, protocol

## Abstract

**Background:**

Many health care facilities in low- and middle-income countries are inadequately resourced. COVID-19 has the potential to decimate surgical health care services unless health systems take stringent measures to protect health care workers from viral exposure and ensure the continuity of specialized care for patients. Among these measures, the timely diagnosis of COVID-19 is paramount to ensure the use of protective measures and isolation of patients to prevent transmission to health care personnel caring for patients with an unknown COVID-19 status or contact during the pandemic. Besides molecular and antibody tests, chest computed tomography (CT) has been assessed as a potential tool to aid in the screening or diagnosis of COVID-19 and could be valuable in the emergency care setting.

**Objective:**

This paper presents the protocol for an umbrella review that aims to identify and summarize the available literature on the diagnostic accuracy of chest CT for COVID-19 in trauma surgery patients requiring urgent care. The objective is to inform future recommendations on emergency care for this category of patients.

**Methods:**

We will conduct several searches in the L·OVE (Living Overview of Evidence) platform for COVID-19, a system that performs automated regular searches in PubMed, Embase, Cochrane Central Register of Controlled Trials, and over 30 other sources. The search results will be presented according to PRISMA (Preferred Reporting Items for Systematic Review and Meta-Analysis). This review will preferentially consider systematic reviews of diagnostic test accuracy studies, as well as individual studies of such design, if not included in the systematic reviews, that assessed the sensitivity and specificity of chest CT in emergency trauma surgery patients. Critical appraisal of the included studies for risk of bias will be conducted. Data will be extracted using a standardized data extraction tool. Findings will be summarized narratively, and the Grading of Recommendations, Assessment, Development, and Evaluation (GRADE) approach will be used to grade the certainty of evidence.

**Results:**

Ethics approval is not required for this systematic review, as there will be no patient involvement. The search for this systematic review commenced in October 2020, and we expect to publish the findings in early 2021. The plan for dissemination is to publish the findings in a peer-reviewed journal and present our results at conferences that engage the most pertinent stakeholders.

**Conclusions:**

During the COVID-19 pandemic, protecting health care workers from infection is essential. Up-to-date information on the efficacy of diagnostic tests for detecting COVID-19 is essential. This review will serve an important role as a thorough summary to inform evidence-based recommendations on establishing effective policy and clinical guideline recommendations.

**Trial Registration:**

PROSPERO International Prospective Register of Systematic Reviews CRD42020198267; https://www.crd.york.ac.uk/PROSPERO/display_record.php?RecordID=198267

**International Registered Report Identifier (IRRID):**

PRR1-10.2196/25207

## Introduction

Many health care facilities in low- and middle-income countries are inadequately resourced. COVID-19 has the potential to decimate their surgical health care services unless health systems take stringent measures to protect health care workers (HCWs) from viral exposure. A recent study showed that 15.6% of patients with confirmed COVID-19 are symptomatic and that nearly half of patients with no symptoms at the time of testing will develop symptoms later [1]. Furthermore, the preoperative evaluation of emergency trauma patients is limited. These factors impede and confound diagnostic triage. Improper infection prevention may create a “superspreader” event in a high-volume health care facility or reduce personnel availability. Consequently, the infection control strategy of trauma surgery staff and in-hospital patients is a top priority for not only low-resource environments but for all emergency trauma facilities with patients presenting with both potential and suspected COVID-19 infection.

In addition to adequate personal protective equipment, appropriate diagnostic testing for patients presenting with an indication for emergency trauma surgery may lead to lower rates of COVID-19 infection among trauma surgery staff and among patients when not isolated. The Prehospital Index (PHI) is a triage-oriented trauma severity scoring system comprising four components: systolic blood pressure, pulse, respiratory status, and level of consciousness, each scored 0 to 5 [2]. A PHI of 4 to 20 indicates major trauma, defined as a patient likely to die within 72 hours after an injury or who requires general or neurosurgical operative intervention within 24 hours. Blunt force trauma, penetrating thoracic and abdominal injuries, severe traumatic brain injury, tension or open pneumothorax, cardiac tamponade, and massive hemothorax are etiologies that will continue to present to emergency departments as indicators for emergency trauma surgery during the COVID-19 period. Time is of the essence for these patients. Thus, guideline recommendations for the diagnostic evaluation for COVID-19 infection must consider time as a resource and allow an evidence-based practice to assuage the cost and benefits of COVID diagnostics for both the patient and for the protection of the trauma surgery staff providing care.

The primary objective of this review is to summarize the diagnostic accuracy of chest computed tomography (CT) imaging for the timely detection of COVID-19, and thus lead to the timely isolation of patients and adequate protection measures to reduce the risk of transmission between patients and the health personnel caring for patients undergoing emergency trauma surgery. The purpose of the review is to inform recommendations for the rational use of chest CT on patients presenting to the emergency department with major trauma, particularly in low-resource environments, where the high costs of the indiscriminate use of diagnostic tools must be avoided without compromising the safety of HCWs or the care of trauma patients. A preliminary search of the International Prospective Registry of Systematic Reviews (PROSPERO), MEDLINE, the Cochrane Database of Systematic Reviews, and the JBI Database of Systematic Reviews and Implementation Reports was conducted, and no current or underway reviews on this topic were identified.

## Methods

### Protocol Registration

The review was registered on PROSPERO (CRD42020198267) and will follow the reporting guidelines of PRISMA (Preferred Reporting Items for Systematic Review and Meta-Analysis). Any changes to the protocol will be amended in PROSPERO and reported in the final review. The authors will include a detailed description of any changes along with a justification during the publication of the review. This review was conducted following the JBI (Joanna Briggs Institute) methodology for systematic reviews [3]. The protocol adheres to the PRISMA guidelines for protocols (PRISMA-P 2015) [4].

### Patient and Public Involvement

Patients and the public were not involved in the design of this umbrella review protocol.

### Study Design

**Figure 1:**
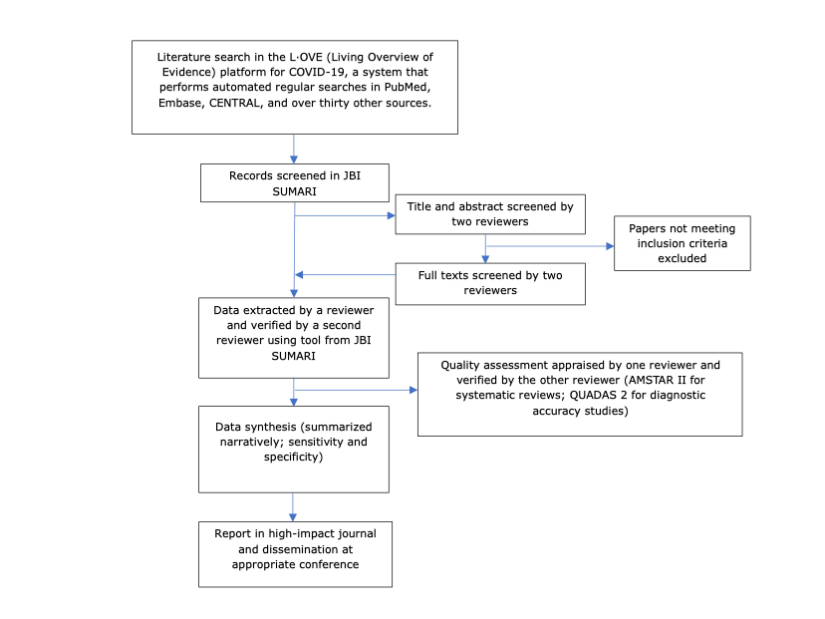
Summary of the review process. CENTRAL: Cochrane Central Register of Controlled Trials; JBI SUMARI: Joanna Briggs Institute System for the Unified Management, Assessment and Review of Information; AMSTAR II: A Measurement Tool to Assess Systematic Reviews II; QUADAS 2: Quality Assessment of Diagnostic Accuracy Studies 2.

A broad evidence synthesis of peer-reviewed and gray literature following the PRISMA approach by Moher et al [5] is planned for this review. Figure 1 summarizes the planned stages of the review as described in this protocol.

### Data Source and Search Strategy

We will conduct several searches in the L·OVE (Living Overview of Evidence) platform for COVID-19, a system that performs automated regular searches in PubMed, Embase, Cochrane Central Register of Controlled Trials (CENTRAL), and over 30 other sources. When compared to manual searches, this platform consistently identifies all the available studies associated with the terms of interest [6-10]. It allows for a fast (automated) search that is easy to update—a crucial element given the urgent need to answer the research question rapidly and thoroughly. We will search for systematic reviews and diagnostic test accuracy (DTA) studies evaluating chest CTs for the diagnosis of COVID-19 in patients presenting with an indication for emergency trauma surgery. Other in-hospital clinical settings will be considered for inclusion and synthesis if evidence for the trauma surgery setting is not available. Different clinical settings will be treated as subgroups from which extrapolation will be possible when considered adequate.

### Selection of Studies

Following the search, all identified citations will be collated and uploaded into EndNote X9 (Clarivate Analytics). The citations will then be imported into JBI SUMARI (Joanna Briggs Institute System for the Unified Management, Assessment and Review of Information) for the review process. Two independent reviewers will examine titles and abstracts for eligibility. The full text of selected studies will be retrieved and assessed. Full-text studies that do not meet the inclusion criteria will be excluded, and a list of such excluded studies will be provided. Disagreements between the reviewers during title and abstract screening or full-text screening will be resolved by consensus or with a third reviewer. The results of the search will be reported in full in the final report and presented via a PRISMA flow diagram [5].

### Eligibility Criteria

#### Inclusion Criteria

##### Participants

The review will preferentially include studies involving emergency trauma surgery patients during the COVID-19 pandemic. Given the likelihood that reports on this specific population are scarce or even nonexistent, if unavailable or insufficient, we will consider studies of patients in any in-hospital setting such as the emergency room, critical care, or general wards, since we consider generalization of such results to be adequate for our question. Studies summarizing the available evidence for other viral respiratory illnesses will not be considered since we do not consider that diagnostic accuracy can be extrapolated to COVID-19.

##### Diagnostic Tests

The diagnostic test under consideration is chest CT for which sensitivity or specificity is assessed.

##### Reference Standard

No individual test is currently considered a true reference (“gold”) standard for COVID-19 diagnosis. We will include studies that used a reference standard of multiple/sequential reverse transcriptase–polymerase chain reaction (RT-PCR), or a composite of viral culture/RT-PCR, and clinical features of COVID-19.

##### Types of Studies

This review will consider systematic reviews of DTA studies and individual DTA studies, if not included in systematic reviews, that fulfill population and diagnostic test criteria. We will also include reports on implementation strategies and costs that could inform recommendations for various resource settings. Only studies published in English or Spanish will be included. We will include preprint studies identified in our search, but no ongoing studies will be considered. Ongoing studies will be counted as excluded studies in the corresponding tables and PRISMA diagram.

#### Exclusion Criteria

We did not identify pertinent exclusion criteria for this review.

### Quality Assessment of Included Studies

Eligible studies will be critically appraised by 2 independent reviewers. We will use the AMSTAR (A Measurement Tool to Assess Systematic Reviews) tool to assess the risk of bias in the systematic reviews, and the QUADAS-2 (Quality Assessment of Diagnostic Accuracy Studies 2) tool for individual diagnostic test accuracy studies [11-13]. The results of the risk of bias assessment will be reported narratively and inform the overall certainty of the review findings. Disagreements will be solved by consensus or by a third reviewer.

### Data Extraction

Data will be extracted from the included studies by a reviewer and verified by a second reviewer using a data extraction tool from JBI SUMARI [3]. The data extracted will include specific details about the populations, study methods, diagnostic tests, diagnostic accuracy, setting, risk of bias of individual studies, and quality of the evidence. Disagreements will be solved by consensus.

### Data Synthesis

Studies will be summarized narratively. Sensitivity and specificity from systematic reviews and from individual studies not included in the systematic reviews will be reported. We do not plan on performing meta-analyses unless we identify primary studies not contained in the included systematic reviews, and such studies are sufficiently homogeneous regarding design, setting, diagnostic tests, and reference standard to consider a meta-analysis adequate. The results for clinically homogeneous studies would be meta-analyzed using RStudio software (RStudio, PBC).

### Assessing Certainty in the Findings

The Grading of Recommendations, Assessment, Development, and Evaluation (GRADE) approach for grading the certainty of evidence will be reported [14,15]. The certainty of findings derived from the individual quality of the systematic reviews and overall consistency of the results will be detailed.

### Data Statement

This review will be based on previously published data. Any relevant data will be published with the review as either an appendix or as an online supplement.

## Results

No ethical approval will be required, as this review is based on already published data and does not involve interaction with human subjects. The search for this systematic review commenced in October 2020, and we expect to publish the findings in early 2021. The plan for dissemination is to publish the review in a peer-reviewed journal and present the findings at high-level international conferences that engage the most pertinent stakeholders.

## Discussion

This protocol has been rigorously developed and designed specifically to identify and summarize the available literature regarding the efficacy of chest CT for patients presenting with an indication for emergency trauma surgery to reduce the risk of COVID-19 infection transmission to the health personnel caring for these patients in low-resource environments. Given the limited recent evidence associated with the primary objective, findings from the review will be critical for researchers, policy makers, and government and nongovernmental organizations for developing recommendations on diagnostic testing for COVID-19 in emergency trauma surgery settings, especially in low- and middle-income countries.

To the best of our knowledge, this protocol provides a detailed description of the first umbrella review on the accuracy of chest CT imaging for the diagnosis of COVID-19 infection. One strength of this research is that it is being conducted by a multidisciplinary team with experience in conducting high-quality evidence synthesis. One limitation is the possibility that new studies will have been published at the time of review publication that were not available at the time of writing the review.

During the COVID-19 pandemic, protecting HCWs from infection is essential and up-to-date information on the accuracy of diagnostic tests for COVID-19 is of great importance.

## Abbreviations

AMSTAR: A Measurement Tool to Assess Systematic Reviews
CENTRAL: Cochrane Central Register of Controlled Trials
CT: computed tomography
DTA: diagnostic test accuracy
GRADE: Grading of Recommendations, Assessment, Development, and Evaluation
HCW: health care worker
JBI: Joanna Briggs Institute
JBI SUMARI: Joanna Briggs Institute System for the Unified Management, Assessment and Review of Information
L·OVE: Living Overview of Evidence
PHI: Prehospital Index
PRISMA: Preferred Reporting Items for Systematic Review and Meta-Analysis
PRISMA-P: Preferred Reporting Items for Systematic Review and Meta-Analysis Protocols
PROSPERO: International Prospective Registry of Systematic Reviews
QUADAS-2: Quality Assessment of Diagnostic Accuracy Studies 2
RT-PCR: reverse transcriptase–polymerase chain reaction
